# Influence of Grout Properties on the Tensile Performance of Rockbolts Based on Modified Cable Elements

**DOI:** 10.3390/ma16155362

**Published:** 2023-07-30

**Authors:** Jianhang Chen, Shiji Wang, Yiqiang Zhao, Lei Liu, Krzysztof Skrzypkowski, Krzysztof Zagórski, Anna Zagórska

**Affiliations:** 1 School of Energy and Mining Engineering, China University of Mining and Technology (Beijing), Beijing 100083, China; wangshiji30@163.com (S.W.);; 2 Faculty of Civil Engineering and Resource Management, AGH University of Science and Technology, Mickiewicza 30 Av., 30-059 Kraków, Poland; 3 Faculty of Mechanical Engineering and Robotics, AGH University of Science and Technology, Mickiewicza 30 Av., 30-059 Kraków, Poland; zagkrzys@agh.edu.pl; 4 Research Centre in Kraków, Institute of Geological Sciences, Polish Academy of Science, Senacka 1, 31-002 Kraków, Poland

**Keywords:** cable element, grout properties, decoupling, in situ stress, rock anchorage, tensile performance

## Abstract

The grout annulus (GA) has a significant effect on the tensile performance of rockbolts in mining engineering. However, little research has been conducted to use modified cable elements to study this effect quantitatively. This paper used the modified cable elements in FLAC3D to study the effect of the GA on the tensile performance of rockbolts. The two-stage coupling law was used to simulate the behaviour of the GA. The stress had a linear relation with the slippage before the shear strength (SS). After the SS, the stress decreased exponentially. Numerical in situ roadway reinforcement cases were used to study the influence of the grout annulus on the tensile performance of rockbolts. The results showed that, when the SS of the GA increased from 3.2 MPa to 6.4 Mpa, the peak force of rockbolts increased from 247 kN to 425 kN. Moreover, when the SS of the GA increased from 3.2 Mpa to 6.4 Mpa, the distance between the position of the maximum tensile capacity and the external end decreased from 1.17 m to 0.81 m. Last, for the circular roadway, the peak force in rockbolts installed in the lateral side was 171.7 kN, which was significantly larger than the top side of 72.3 kN.

## 1. Introduction

Rockbolts are cylindrical bars that are installed into rock masses to improve the stability of underground openings in mining [1,2,3]. In the rockbolting system, there are mainly three components: rockbolts, grout and rock masses. Among them, the grout is the medium that connects rockbolts and rock masses [4,5,6].

In rock reinforcing in mining, two different types of grouts were used: resin-based grout and cement-based grout [7]. In situ cases demonstrate that grout properties significantly affect the tensile performance of rockbolts [8]. Moreover, the tensile performance of rockbolts will affect the stability of underground openings [9,10].

In situ tests demonstrated that, after rockbolts are installed around the underground openings, parameters affected the stability of underground openings, including the grout strength, the spacing between rockbolts, the installation angle of rockbolts, and the rockbolt length [11].

To disclose the influence of grout properties on the tensile performance of rockbolts, a number of experimental studies have been conducted [12]. The most commonly used experimental method is to conduct the tensile tests on the rockbolting system [13]. The rockbolt will be installed into natural or artificial rock masses. Then, the grout is used to bond the rockbolt and the rock masses. After that, a hydraulic cylinder is used to pull the rockbolt from the rock masses. Meantime, the pull force and displacement are recorded to reflect the tensile performance of rockbolts [14]. Additionally, researchers may attach strain gauges on rockbolts to measure the force state on rock bolts [15].

Kilik, et al. [16] conducted around eighty pull tests on rockbolts. They found that the water/cement (w/c) ratio of the cement-based grout influenced the tensile performance of rockbolts. They recommended that the optimised w/c rate for the rockbolting system was between 0.35 and 0.4. Following this, Chen, et al. [8] studied the influence of w/c ratio of the grout on the grout strength. They especially focused on optimising the w/c ratio that would be used in rockbolt installation. Kılıc, et al. [17] conducted pull tests on fully grouted rockbolts. They found that grout with high strength generated a wedging effect. Moreover, this wedging effect improved the tensile performance of rockbolts. Chen and Mitri [18] compared the tensile performance of grouted cablebolts with different grout strength. Two different w/c rates were used: 0.3 and 0.4. The results showed that, with the w/c rate increasing, the stiffness of the cable/grout joint declined dramatically.

Mosse-Robinson and Sharrock [19] studied the influence of grout strength on the tensile performance of bulbed cablebolts. The cement-based grout was used as the bonding agent with the w/c rate changing between 0.35 and 0.45. They found that the tensile performance of bulbed cablebolts had an adverse relation with the w/c rate. Li, et al. [20] evaluated the tensile performance of rockbolts when the grout strength was different. They found that the bond strength of the rockbolting system had a linear relation with the grout strength. Teymen and Kilic [21] attached strain gauges on rockbolts and conducted pull tests on rockbolts. They evaluated the influence of grout properties on the stress distribution in the rockbolting system. The results showed that with the grout strength increasing, the stress distribution tended to become uniform.

Li [22] conducted double embedment pull tests on cablebolts. The influence of grout age on the tensile performance of cablebolts was studied. They found that the grout age apparently influenced the maximum tensile capacity of rockbolts. However, the grout age had little influence on the force–displacement relation of cablebolts. Wang, et al. [23] found that the peak force of rockbolts was influenced by the grout strength. With the grout strength increasing, the peak force increased. Yu, et al. [2] conducted pull tests on rockbolts. They found that, when the rockbolt diameter was constant, the peak force increased with the cement-based grout strength. The corresponding displacement where the peak force generated also increased. However, the cement-based grout strength had no direct impact on the residual pull force of rockbolts. Høien, et al. [9] adjusted the w/c rate of the grout to change the grout strength. They studied the tensile performance of rockbolts when the grout strength was different. The results showed that the grout strength influenced the critical grouted length.

Comparatively, much less work has been conducted on using the numerical method to study the influence of grout properties on the tensile performance of rockbolts. More attention was paid to using the numerical method to study the rock reinforcing performance of rockbolts under the in situ condition [24,25,26,27,28].

The advantage of using the numerical simulation method includes the ability to use it to predict the tensile performance of rockbolts under different rock reinforcement scenarios. Therefore, in this study, the authors tried to use the numerical simulation method to study the influence of the GA on the tensile performance of rockbolts.

In numerical simulating, the structure elements of cable in FLAC3D are commonly used. The advantage of this is that the cable elements can simulate the rock reinforcing tendons including rockbolts and cablebolts. However, the shortcoming is that, in the original cable elements, the spring slider system used a perfectly plastic model to simulate the load transfer between rock reinforcing tendons and rock masses. Therefore, it cannot truly reflect the anchorage performance of rock tendons.

In this study, the modified cable elements in FLAC3D were used to simulate rockbolts. The two-stage coupling law was used to simulate the bolt/grout joint. The stress had a linear relation with the slippage before the shear strength (SS). After the SS, the stress decreased exponentially.

Numerical pull tests were conducted to confirm the credibility of the modified cable elements. Then, the influence of grout properties on the tensile performance of rockbolts under the numerical pull test scenario and in situ roadway reinforcement scenario was studied.

## 2. Materials and Methods

### 2.1. Two-Stage Coupling Law

**Figure 1:**
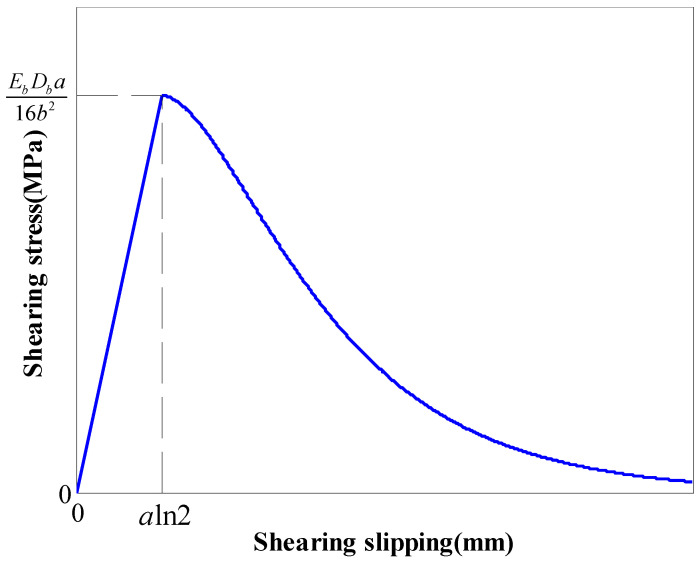
Relation between the stress and the slippage.

A two-stage coupling law was used to simulate the grout annulus (GA). In the elastic stage, the stress increases linearly to the SS with the slippage, as calculated with Equation (1). Then, the stress decreases exponentially [29], as calculated with Equation (2), as shown in Figure 1.

(1) $$\tau =\frac{E_{b}D_{b}}{16b^{2}\ln2}s\;(s\text{≤}a\ln2)$$

(2) $$\tau =\frac{E_{b}D_{b}}{4}\frac{a}{b^{2}}e^{-\frac{s}{a}}\left(1-e^{-\frac{s}{a}})(s\text{>}a\ln2\right)$$

where *τ*: shear stress of the GA; *E_b_*: rockbolt modulus; *D_b_*: rockbolt diameter; *s*: slippage of the GA; *a*, *b*: coefficients.

Following this law, before the shear stress of the grout annulus reached the SS, the grout annulus was intact. After the SS, the grout annulus started damaging.

There were two coefficients, *a* and *b*, which had a direct impact on the behaviour of the GA. To reveal the influence of these two coefficients on the behaviour of the GA, a parametric study was conducted.

**Figure 2:**
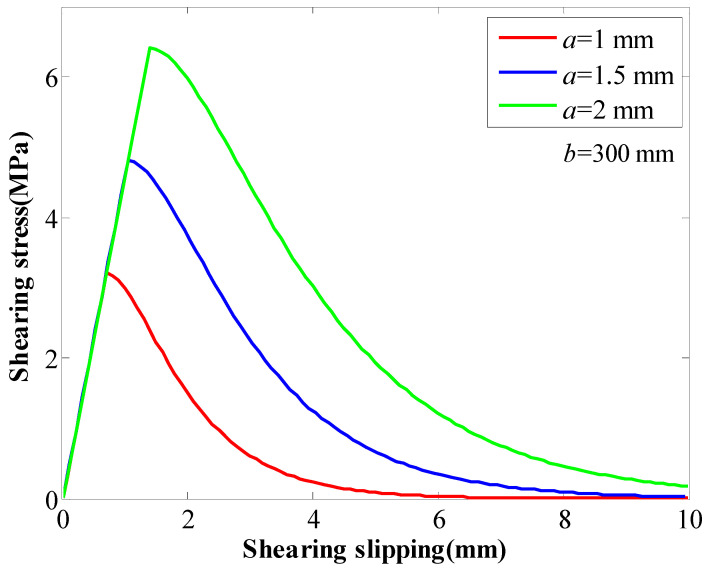
The influence of coefficient *a* on the behaviour of the GA.

The tested rockbolt had a modulus of 210 GPa and diameter of 22 mm. The coefficient *b* was 300 mm. The coefficient *a* changed from 1 mm to 2 mm. With the coefficient *a* increasing, the SS of the GA increased from 3.2 MPa to 6.4 MPa (Figure 2). Moreover, the coefficient *a* had an impact on slippage when the SS was reached. With the SS of the GA increasing, the slippage when the SS increased from 1 mm to 1.4 mm.

**Figure 3:**
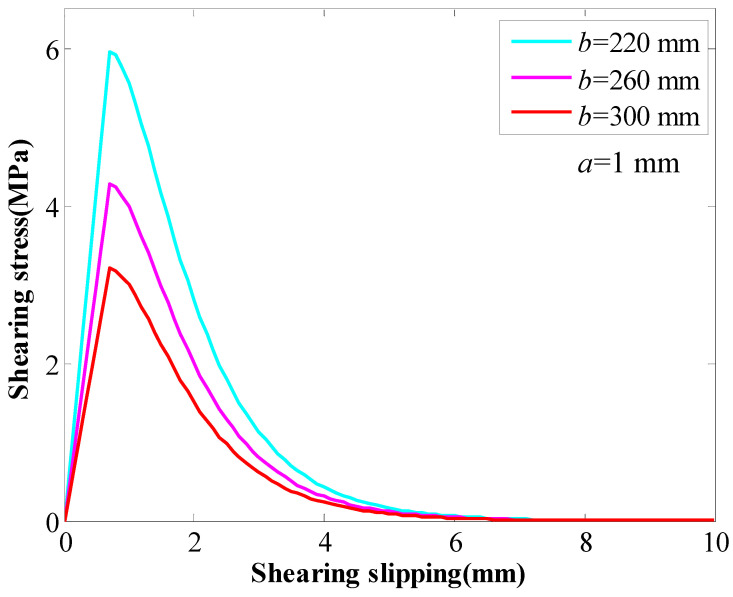
The influence of coefficient *b* on the behaviour of the GA.

The other parametric study was conducted to study the influence of the coefficient *b* on the behaviour of the GA. The coefficient *a* was 1 mm. The coefficient *b* changed between 220 mm and 300 mm. With the coefficient *b* increasing, the SS and the stiffness of the GA decreased continuously (Figure 3). With the coefficient *b* increasing from 220 mm to 300 mm, the SS of the GA decreased from 6.0 MPa to 3.2 MPa.

### 2.2. Modified Cable Elements

The cable elements are one kind of structure element embedded in FLAC3D (FLAC3D 6.0, Itasca company, Minneapolis, MN, USA). The cable elements are composed of several segments and nodes. The mechanical properties of the GA are incorporated in these nodes. These nodes interact with the surrounding numerical zones, which then simulate the rock masses. Therefore, the interaction between the rock reinforcing tendons and the rock masses can be simulated.

However, the original cable elements used a perfectly plastic model to simulate the behaviour of the GA, which cannot truly reflect the interaction between rock reinforcing tendons and the rock masses. Therefore, this study modified the original cable elements.

For the cable elements, when the friction angle of the GA is fixed to zero, the bond strength of the GA can be adjusted by the cohesive strength of the GA manually [30], as calculated with Equation (3):

(3) $$\frac{F_{s}}{L}=c_{g}$$

where *F_s_*: shear force in the GA; *L*: length of the segment; and *c_g_*: cohesive strength of the GA in a unit length.

The relation between the cohesive strength of the GA in a unit length and the stress of the GA can be written as:

(4) $$c_{g}=\tau \pi D_{b}$$

Substituting Equations (1) and (2) into Equation (4) leads to:

(5) $$c_{g}=\frac{E_{b}D_{b}}{16b^{2}\ln2}s\pi D_{b}$$

(6) $$c_{g}=\frac{E_{b}D_{b}}{4}\frac{a}{b^{2}}e^{-\frac{s}{a}}\left(1-e^{-\frac{s}{a}}\right)\pi D_{b}$$

Based on Equations (5) and (6), the two-stage coupling law can be incorporated into the cable elements.

To realise the above process, a FISH function “incorporating” was created in FLAC3D. The FISH function was set by FLAC3D. It created a link between the user and the functions of nodes, zones and structure elements. Users can then require the nodes, zones and structure elements to operate based on the user-defined rules.

The FISH function of “incorporate” was utilized in each step during the entire calculation process. During the calculation, this FISH function checked the grout displacement at each node in the cable elements. Then, it substituted the grout displacement into the two-stage coupling law to calculate the stress. Finally, the calculated stress was updated at each node in the cable elements.

Credibility of the modified cable elements has been validated with experimental tests in the previous paper written by the authors. The validation process indicated that the modified cable elements were robust in studying the performance of rockbolts.

### 2.3. Numerical Pull Tests

To check the credibility of the modified cable elements, numerical pull tests were conducted. The numerical pull test scenario is listed as below. A rockbolt with a diameter of 22 mm was embedded in the numerical rock block. The modulus of the rockbolt is 210 GPa and the grouted length is 1 m.

For the numerical rock block, a Mohr–Coulomb model was used as the constitutive law. The mechanical properties of the numerical rock block are listed in Table 1.

At the loaded end, the front surface of the numerical rock block was fixed. Therefore, no movement of the front surface was allowed. After that, the modified cable elements were installed in the middle of the numerical rock block. As for the mechanical properties of modified cable elements, they were listed as below: modulus 210 GPa, exposed perimeter of the grout 69.12 mm, cross-sectional area of the rockbolt 380.13 mm^2^. The stiffness of the GA is calculated with Equation (7):

(7) $$k_{g}=\frac{E_{b}\pi D_{b}^{2}}{16\ln2b^{2}}$$

where *k_g_*: stiffness of the GA.

#### 2.3.1. Numerical Pull Tests when the Coefficient of *a* Varied

The first series of numerical pull tests was conducted when the coefficient of *a* varied. In this case, the coefficient of *b* was fixed to 300 mm. The coefficient of *a* varied from 1 mm to 2 mm. During the pull process, a small pull velocity of 1 × 10^−6^ m/s was applied at the loaded end of the cable element. In the numerical pull test, the pull force and displacement were recorded to reflect the tensile performance of rockbolts.

Moreover, during the numerical pull process, the behaviour of the GA was recorded. Specifically, the relation between the stress and the slippage of the GA was recorded. The purpose of this recording is to confirm whether the two-stage coupling law has been incorporated into the cable elements.

#### 2.3.2. Numerical Pull Tests when the Coefficient of *b* Varied

The second series of numerical pull tests was conducted when the coefficient of *b* varied. In this case, the coefficient of *a* was fixed to 1 mm. Then, the coefficient of *b* varied from 220 mm to 300 mm.

### 2.4. Numerical In Situ Roadway Reinforcement Tests

In the above analysis, numerical pull tests were conducted on modified cable elements. However, in this analysis, the in situ stress in the rock masses was not considered. Therefore, in this section, the modified cable elements were installed in the rock masses around the numerical roadway under the in situ stress condition.

**Figure 4:**
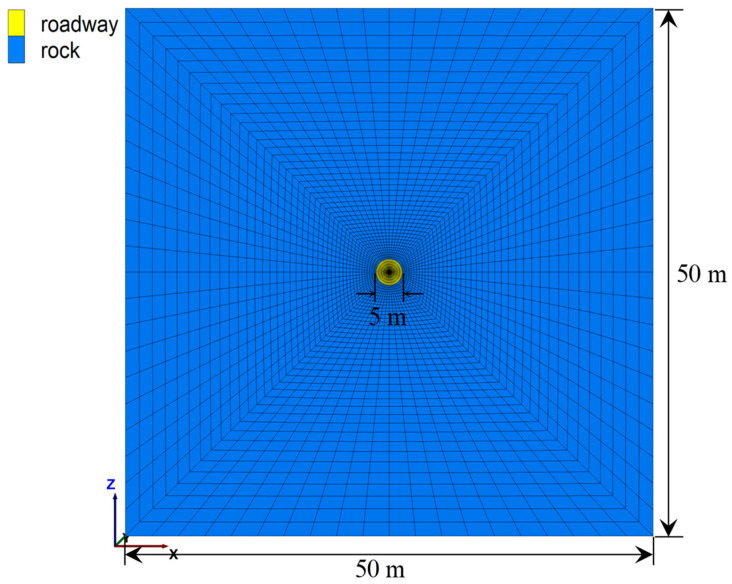
Numerical roadway excavation scenario.

Specifically, a numerical mesh whose width and height were 50 m was created (Figure 4). The thickness of this numerical mesh was 1 m. In the middle of this numerical mesh, a circular roadway whose diameter was 5 m was excavated.

To simulate the rock masses, the strain-softening model was used. The mechanical properties of the rock masses are listed in Table 2.

To simulate the strain-softening behaviour of rock masses, a cohesion table was used. This cohesion table defined that, when the plastic shear strain was 0, the cohesion was 2.5 MPa. When the plastic shear strain increased to 1 × 10^−4^, the cohesion decreased to 2 MPa. When the plastic shear strain continuously increased to 1.5 × 10^−4^, the cohesion decreased to 1.5 MPa.

For the boundary conditions, the left side, the right side, the internal side, and the external side of the numerical mesh were set as roller boundaries. As for the bottom side, the fixed boundary was set. Then, for the top side, a vertical compressive stress of 15 MPa was added.

For this numerical mesh, a density of 2300 kg/m^3^ was set. Then, the initial stress of the numerical mesh was generated by the gravity action.

**Figure 5:**
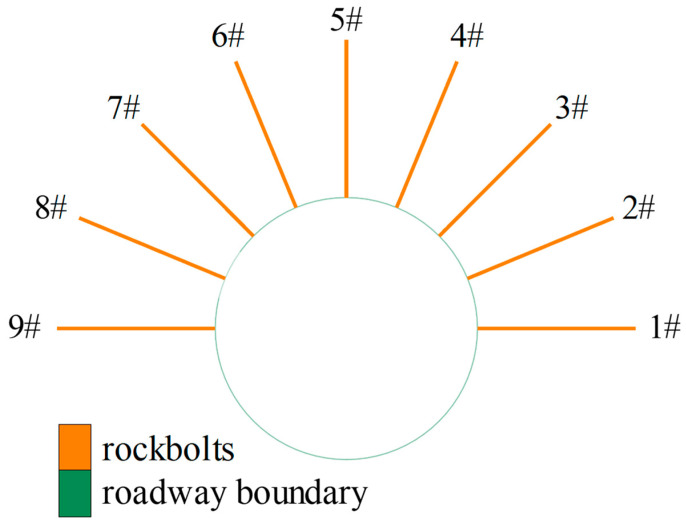
Simulating rockbolts with modified cable elements around the roadway.

After the initial stress equilibrium was achieved, the roadway was excavated. After that, nine rockbolts with a length of 3 m were installed with the modified cable elements used (Figure 5).

The mechanical properties of the rockbolts are listed in Table 3. The stiffness of the GA was calculated with Equation (7).

#### 2.4.1. Numerical Rock Reinforcing When the Coefficient of *a* Varied

Three numerical cases were calculated. In these three numerical cases, the coefficient of *b* was fixed to 300 mm. As for the coefficient of *a*, it varied from 1 mm to 2 mm. Then, the automatic calculation was conducted until the average unbalanced force ratio decreased to 1 × 10^−5^. After the numerical mesh reaches the equilibrium, the force distribution along rockbolts was recorded to check the tensile performance of rockbolts.

#### 2.4.2. Numerical Rock Reinforcing When the Coefficient of *b* Varied

Another three numerical cases were conducted to study the tensile performance of rockbolts when the coefficient of *b* varied. In these three numerical cases, the coefficient of *a* was fixed to 1 mm. Then, the coefficient of *b* varied from 220 mm to 300 mm. In all these three numerical cases, the automatic calculation was conducted until the average unbalanced force ratio decreased to 1 × 10^−5^.

## 3. Results

### 3.1. Numerical Pull Test Results

#### 3.1.1. Numerical Pull Test Results When the Coefficient of *a* Varied

**Figure 6:**
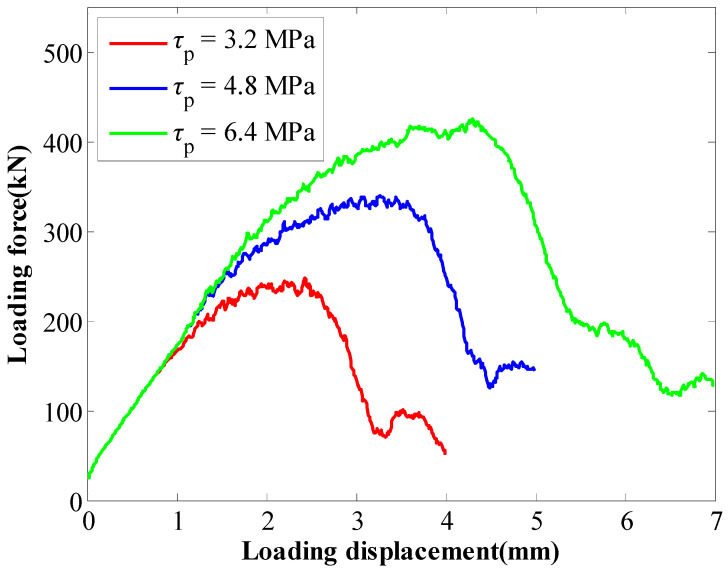
Tensile performance of rockbolts when the coefficient of *a* varied.

For the numerical pull tests, when the coefficient of *a* varied from 1 mm to 2 mm, the SS of the GA (*τ_p_*) increased from 3.2 MPa to 6.4 MPa. The tensile performance of rockbolts is shown in Figure 6. It shows that when the SS of the GA increased, the peak force of rockbolts increased from 247 kN to 425 kN. Moreover, the displacement where the peak force generated also increased. In this case, the displacement where the peak force generated increased from 2.43 mm to 4.3 mm. The results indicated that the SS of the GA had a direct relation on the tensile performance of rockbolts. The larger the SS of the GA, the higher the peak force of rockbolts.

**Figure 7:**
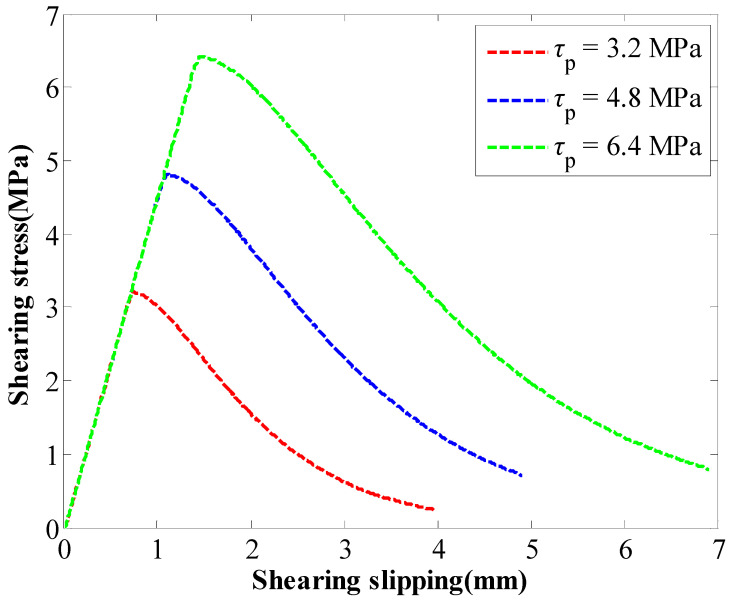
Exported results between the stress of the GA and the slippage.

In this numerical pull process, the exported results between the stress and slippage of the GA are shown in Figure 7. Apparently, the original perfectly plastic law has been modified. When the stress of the GA reached the SS, it decreased in an exponential way.

Comparing Figure 2 and Figure 7 indicated that there was a good consistence between them. This indicated that the two-stage coupling law was successfully incorporated into the cable elements.

#### 3.1.2. Numerical Pull Test Results When the Coefficient of *b* Varied

**Figure 8:**
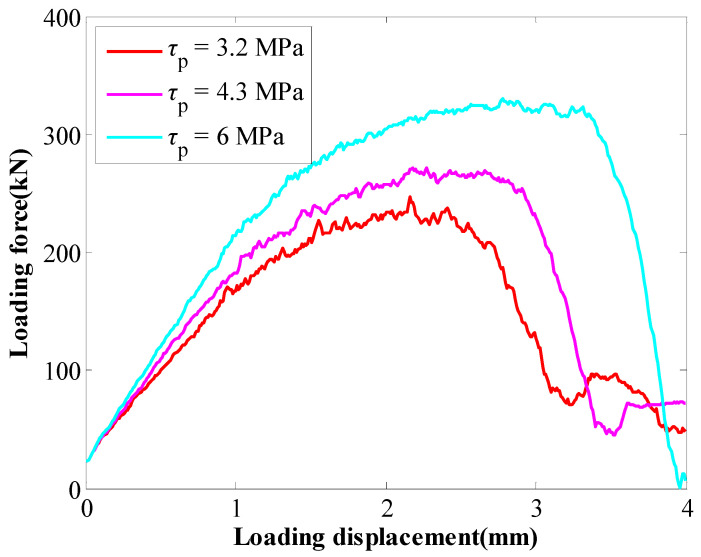
Tensile performance of rockbolts when the coefficient of *b* varied.

When the coefficient of *b* varied from 220 mm to 300 mm, the SS of the GA decreased from 6 MPa to 3.2 MPa. The tensile performance of rockbolts is shown in Figure 8. The results further confirmed that the SS of the GA directly affected the tensile performance of rockbolts. With the SS of the GA decreasing from 6 MPa to 3.2 MPa, the peak force of rockbolts decreased from 330 kN to 246 kN. Furthermore, the displacement where the peak force reached also decreased from 2.78 mm to 2.16 mm.

**Figure 9:**
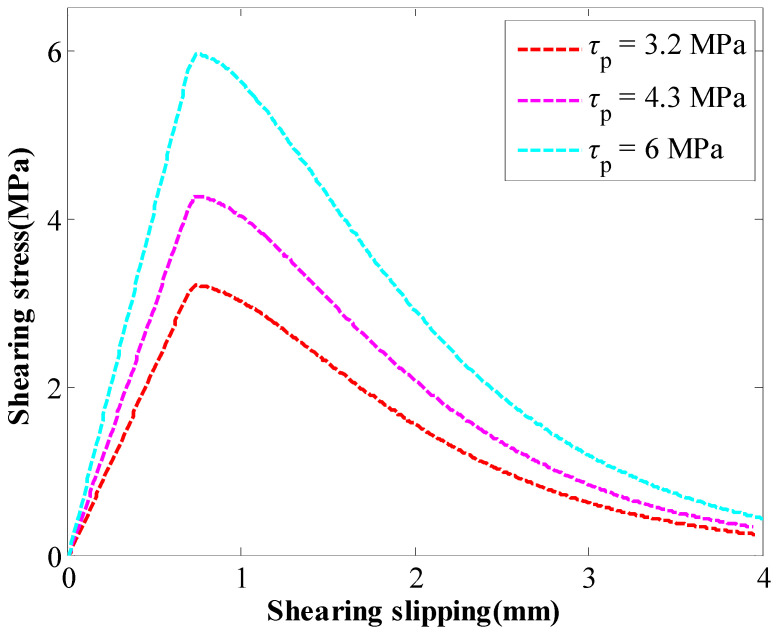
Exported results between the stress of the GA and the slippage.

In this numerical pull case, the behaviour of the GA was exported (Figure 9). Apparently, the original perfectly plastic model in the cable element was revised. When the stress of the GA reached the SS, it decreased exponentially.

A comparison was conducted between Figure 3 and Figure 9. Apparently, a good correlation between them occurred. This indicated that the proposed two-stage coupling law was successfully incorporated into the cable elements.

### 3.2. Numerical In Situ Roadway Reinforcement Results

#### 3.2.1. Numerical Rock Reinforcing Results When the Coefficient of *a* Varied

**Figure 10:**
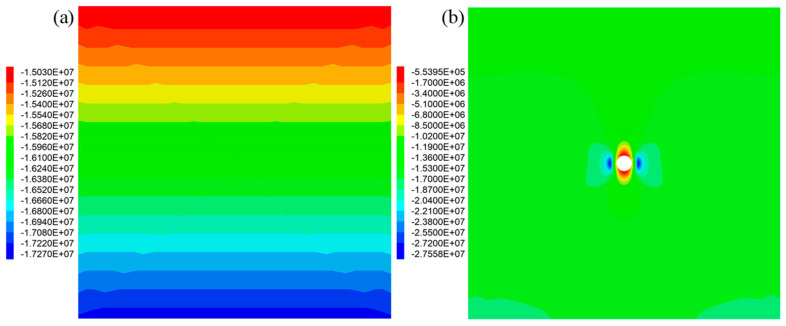
Vertical stress distribution in the numerical mesh: (**a**) initial vertical stress after the equilibrium was reached; (**b**) vertical stress distribution after the roadway was excavated.

For the numerical in situ roadway reinforcement cases, after the initial stress equilibrium was reached, the minimum vertical stress was around 15 MPa and the maximum vertical stress was 17.3 MPa, as shown in Figure 10a.

According to the Heim’s law, the buried depth can be calculated based on Equation (8).

(8) $$\sigma_{zz}=\rho gH$$

where *σ*_*zz*_: vertical stress in the numerical zone; *ρ*: density of the rock masses; *g*: gravitational acceleration; *H*: buried depth.

It is assumed that the average density of the rock masses was 2500 kg/m^3^. Substituting this into Equation (8), the buried depth in this case ranged from 600 m to 692 m.

After the roadway was excavated, the vertical stress distribution is shown in Figure 10b. Apparently, the roadway excavation significantly affected the vertical stress distribution in the rock masses. At the left side and right side of the roadway, the vertical stress significantly increased to 27.6 MPa. As for the top side and bottom side of the roadway, the vertical stress decreased to around 0.6 MPa.

**Figure 11:**
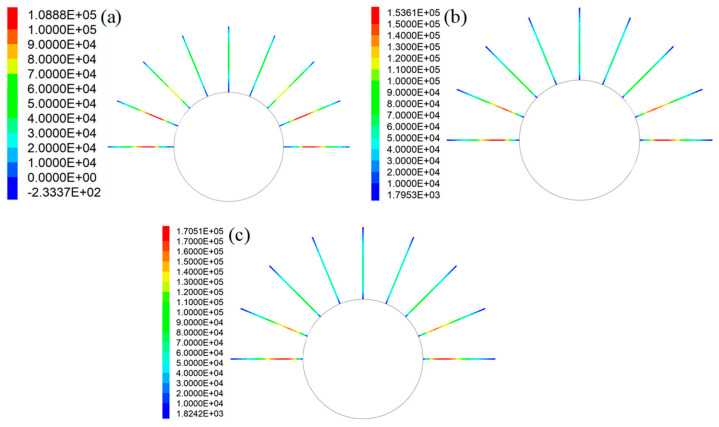
Tensile force distribution in rockbolts when the coefficient of *a* varied: (**a**) *τ_p_* = 3.2 MPa; (**b**) *τ_p_* = 4.8 MPa; (**c**) *τ_p_* = 6.4 MPa.

This study mainly focused on the influence of grout properties on the tensile performance of rockbolts. Therefore, in this case, the tensile force distribution in rockbolts is plotted (Figure 11). Apparently, when the coefficient of *a* varied, the SS of the GA changed from 3.2 MPa to 6.4 MPa. Consequently, the tensile force distribution of the rockbolts also changed.

**Figure 12:**
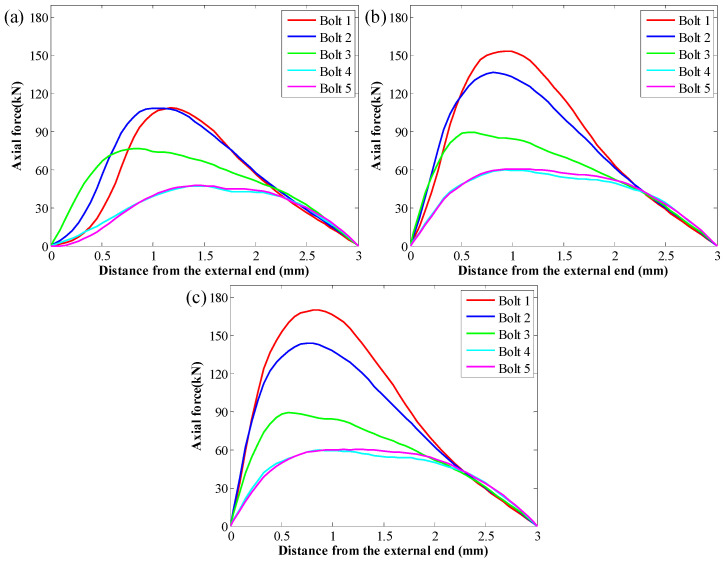
Tensile force distribution trend in rockbolts when the coefficient of *a* varied: (**a**) *τ_p_* = 3.2 MPa; (**b**) *τ_p_* = 4.8 MPa; (**c**) *τ_p_* = 6.4 MPa.

When the SS of the GA was 3.2 MPa, the peak force in rockbolts was 108.9 kN. Then, when the SS of the GA was 4.8 MPa, the peak force in rockbolts was 153.6 kN. When the SS of the GA was 6.4 MPa, the peak force in rockbolts was 170.5 kN. Therefore, under the same in situ stress condition, there was a positive relation between the peak force in rockbolts and the SS of the GA. Additionally, the SS of the GA had no apparent influence on the tensile force distribution trend of rockbolts. This is more apparent when the tensile force distribution trend was plotted (Figure 12). Since the rockbolt reinforcement and the numerical model were symmetrical along the central vertical line, the tensile force distribution of five rockbolts at the right side was plotted.

The results show that although the SS of the GA was different, the tensile force distribution trend in rockbolts was similar. The tensile force was maximal around the middle section of rockbolts. By contrast, the tensile force was minimal at two ends of rockbolts.

It is also found that the SS of the GA affected the position of the peak force in rockbolts. In this case, with the SS of the GA increasing from 3.2 MPa to 6.4 MPa, the position of the peak force in rockbolts moved towards the external end of rockbolts. The rockbolt 1# was taken as an example. When the SS of the GA was 3.2 MPa, the distance between the position of the maximum tensile capacity and the external end was 1.17 m. Then, when the SS of the GA was 4.8 MPa, the distance between the position of the peak force and the external end was 0.93 m. When the SS of the GA was 6.4 MPa, the distance between the position of the maximum tensile capacity and the external end was 0.81 m.

Additionally, in all three cases, the tensile force in rockbolts installed in the lateral side was larger than the tensile force in rockbolts installed in the top side. When the SS of the GA was 3.2 MPa, the peak force in rockbolts installed in the lateral side was 108.9 kN. The peak force in rockbolts installed in the top side was 47.6 kN. When the SS of the GA was 4.8 MPa, the peak force in rockbolts installed in the lateral side was 153.6 kN. The peak force in rockbolts installed in the top side was 60.4 kN. When the SS of the GA was 6.4 MPa, the peak force in rockbolts installed in the lateral side was 170.5 kN. The peak force in rockbolts installed in the top side was 60.2 kN.

#### 3.2.2. Numerical Rock Reinforcing Results When the Coefficient of *b* Varied

**Figure 13:**
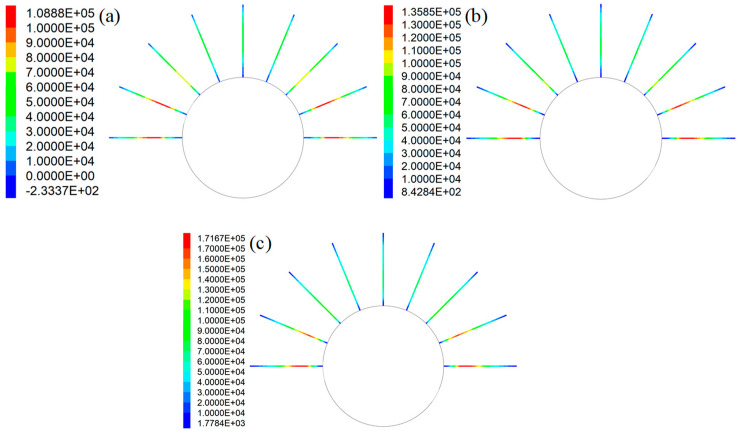
Tensile force distribution in rockbolts when the coefficient of *a* varied: (**a**) *τ_p_* = 3.2 MPa; (**b**) *τ_p_* = 4.3 MPa; (**c**) *τ_p_* = 6 MPa.

When the coefficient of *b* varied from 220 mm to 300 mm, the SS of the GA decreased from 6.0 MPa to 3.2 MPa. After the roadway was excavated, the tensile force distribution along rockbolts is shown in Figure 13. Apparently, the SS of the GA significantly affected the force distribution along rockbolts. The larger the SS of the GA, the higher the peak force of rockbolts. When the SS of the GA was 3.2 MPa, the peak force of rockbolts was 108.9 kN. When the SS of the GA was 4.3 MPa, the peak force of rockbolts was 135.9 kN. When the SS of the GA was 6 MPa, the peak force of rockbolts was 171.7 kN. This demonstrated that improving the SS of the GA was likely to increase the peak force of rockbolts.

**Figure 14:**
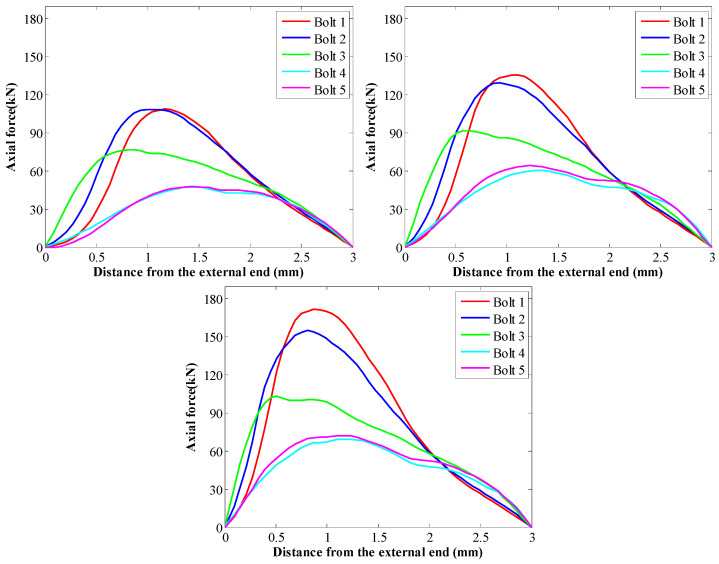
Tensile force distribution trend in rockbolts when the coefficient of *b* varied: *τ_p_* = 3.2 MPa; *τ_p_* = 4.3 MPa; *τ_p_* = 6 MPa.

To further study the influence of the GA on the force distribution in rockbolts, the tensile force trend is plotted (Figure 14). It shows that the SS of the GA had no impact on the tensile force distribution trend. Although the SS of the GA was different, the tensile force was maximal around the middle of rockbolts. By contrast, the minimum tensile force was generated at two ends of rockbolts.

The results showed that the SS of the GA affected the position where the peak force was generated. The rockbolt 1# was taken as an example. When the SS of the GA was 3.2 MPa, the distance between the position where the peak force was generated and the external end was 1.17 m. When the SS of the GA was 4.3 MPa, the distance between the position where the peak force was generated and the external end was 1.11 m. When the SS of the GA was 6 MPa, the distance between the position where the peak force was generated and the external end was 0.87 m. Therefore, with the SS of the GA increasing, the peak force in rockbolts moved towards the external end of rockbolts.

Additionally, in all these three cases, the tensile force in rockbolts installed in the lateral side of roadways was larger than the tensile force in rockbolts installed in the top side of roadways. When the SS of the GA was 3.2 MPa, the peak force in rockbolts installed in the lateral side was 108.9 kN. The peak force in rockbolts installed in the top side was 47.6 kN. When the SS of the GA was 4.3 MPa, the peak force in rockbolts installed in the lateral side was 135.8 kN. The peak force in rockbolts installed in the top side was 64.3 kN. When the SS of the GA was 6 MPa, the peak force in rockbolts installed in the lateral side was 171.7 kN. The peak force in rockbolts installed in the top side was 72.3 kN.

## 4. Discussion

This paper used the modified cable elements to simulate rockbolts and studied the tensile performance of rockbolts. Two different scenarios were used. One was the numerical pull test scenario. The other one was the numerical in situ roadway reinforcement scenario.

In the numerical pull test scenario, when the SS of the GA increased, the peak force of rockbolts increased. This finding agrees well with the experimental test conducted by Kilik, et al. [16]. In the experimental pull tests on rockbolts, Kilik, et al. [16] found that when the shear strength of the cement-based grout increased from 2.04 MPa to 11.93 MPa and the peak force of rockbolts increased from 16.53 kN to 80.87 kN. Therefore, there was a positive relationship between the shear strength of the grout and the peak force of rockbolts. This further confirmed the credibility of this numerical simulation result.

The reason for this result is that the SS of the GA represented the bearing ability of the GA. When the SS of the GA increased, the GA can bear larger shear stress.

This study focused on the static tensile performance of rockbolts. Therefore, there was an equilibrium between the shear stress in the GA and the tensile stress in the rockbolt. Consequently, when the SS of the GA increased, the peak force of rockbolts also increased.

This phenomenon also occurred in the numerical in situ roadway reinforcement scenario. In this case, when the SS of the GA increased from 3.2 MPa to 6.4 MPa, the peak force in rockbolts consistently increased from 108.9 kN to 170.5 kN. This was also because when the SS of the GA was larger, it indicated that the GS could bear higher shear stress. Since there was an equilibrium in the rockbolting system, higher shear stress in the GA led to larger peak force in rockbolts.

**Figure 15:**
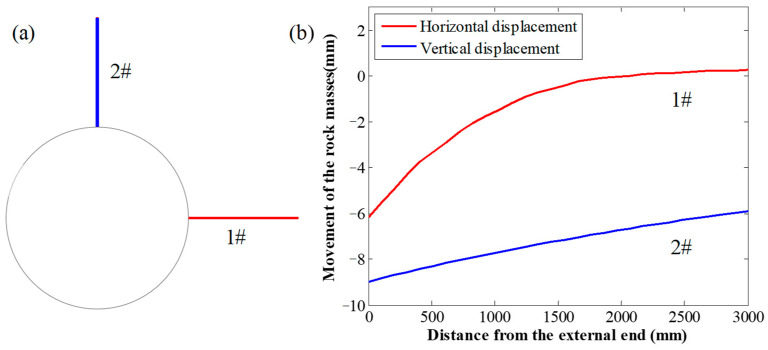
Rock mass displacement recorded by the measuring line: (**a**) location of the measuring line; (**b**) rock mass displacement distribution.

The results also indicated that, although the SS of the GA was different, the tensile force in rockbolts installed in the lateral side of roadways was consistently larger than the tensile force in roadways installed in the top side of roadways. To analyse this reason, when *a* = 1 mm and *b* = 300 mm, two measuring lines with a length of 3 m were set in the numerical mesh, as shown in Figure 15a. The measuring line 1# was used to record the horizontal displacement of the rock masses, and the measuring line 2# was used to record the vertical displacement of the rock masses.

The recorded results are shown in Figure 15b. It shows that, along the lateral side of the roadway, the relative displacement of the rock masses at the two ends of the measuring line was 6.42 mm. By contrast, along the top side of the roadway, the relative displacement of the rock masses at the two ends of the measuring line was 3.1 mm.

Therefore, compared with the top side of the roadway, the relative displacement of the rock masses at two ends of the measuring line was doubled when the rockbolt was installed in the lateral side of the roadway. This indicated that there was a significant difference on the relative difference of the rock masses at different locations.

Moreover, it indicated that, under the in situ vertical stress condition, the relative horizontal displacement of the rock masses along the lateral side of the roadway was larger than the relative vertical displacement of the rock masses along the top side of the roadway. Therefore, along the lateral side of the roadway, after the rockbolt was installed, this relative larger displacement of rock masses can induce higher stress in the GA.

This study focused on the static loading performance of rockbolts. Therefore, there was an equilibrium between the shear stress in the GA and the tensile stress in the rockbolt. Then, higher stress in the GA meant that larger stress was induced in the rockbolt.

Consequently, the tensile force in rockbolts installed in the lateral side of the roadway was larger than the tensile force in rockbolts installed in the top side of the roadway.

**Figure 16:**
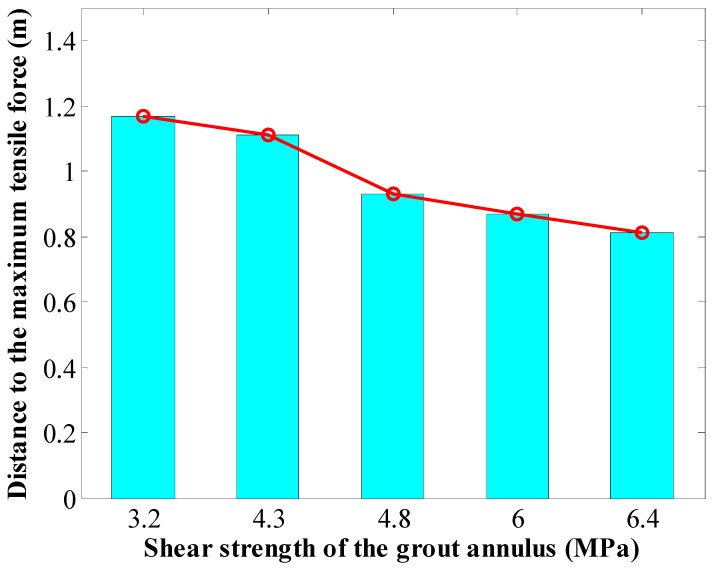
Distance to the peak force when the SS of the GA varied.

It is also found that, under the numerical in situ roadway reinforcement scenarios, with the SS of the GA increasing, the position where the peak force of rockbolts moved outwards. For the rockbolt 1#, when the SS of the GA increased from 3.2 MPa to 6.4 MPa, the distance between the external end to the position where the peak force generated decreased from 1.17 m to 0.81 m (Figure 16).

The reason for this result is that, when the SS of the GA was small, the stress in the GA in the external section was not large enough to reach the equilibrium along the longitudinal direction of rockbolts. Therefore, the stress in the GA was mobilised towards the internal section. Consequently, in this case, the maximum tensile force of rockbolts was generated relatively far away from the external end of rockbolts.

By contrast, when the SS of the GA was large, the stress in the GA in the external section was large enough to maintain the equilibrium along the longitudinal direction of rockbolts. Therefore, not enough stress was transferred to the internal section of rockbolts. Consequently, the peak force of rockbolts was generated around the external end of rockbolts.

This study indicated that using modified cable elements to study the tensile performance of rockbolts was reliable. Therefore, in practical engineering, the modified cable elements can be used to pre-calculate the tensile performance of rockbolts. This can provide a theoretical foundation for optimising the design of rockbolts.

## 5. Conclusions

The grout annulus (GA) had a significant impact on the tensile performance of rockbolts. However, until now, little research has been conducted to study the influence of GA on the tensile performance of rockbolts with modified cable elements. Therefore, this study modified the original cable elements in the FLAC3D and adopted the modified cable elements to study the influence of GA on the tensile performance of rockbolts.

(1) The numerical pull tests indicated that the peak force of rockbolts increased with the shear strength (SS) of the GA increasing. Moreover, the corresponding pull displacement at the peak force increased.

(2) A FISH function was created to modify the original cable elements. The results showed that, based on the proposed FISH function, the two-stage coupling law can be successfully incorporated into the cable elements.

(3) The SS of the GA significantly affected the peak force in rockbolts. The larger the SS of the GA, the higher the peak force in rockbolts. The position where the peak force generated moved outwards when the SS of the GA increased.

(4) The methods used in this paper can be used to predict the tensile performance of rockbolts before mining activities are conducted in underground mines. Therefore, they can be used to optimise the design of the rockbolt reinforcement systems

## 6. Recommendation for Further Research

Further work may continue to optimise the grout ingredients to improve the tensile performance of rockbolts. Moreover, new additives may be added to reduce the cost.

## Figures and Tables

**Table 1:** Properties of the numerical rock block.

Modulus	Poisson’s Ratio	Tensile Strength	Friction Angle	Cohesion
15 GPa	0.25	2 MPa	32°	3 MPa

**Table 2:** Properties of the numerical mesh.

Modulus	Poisson’s Ratio	Tensile Strength	Friction Angle	Cohesion
11 GPa	0.25	1.2 MPa	32°	2.5 MPa

**Table 3:** Properties of the numerical rockbolts.

Modulus	Exposed Perimeter of the GA	Cross-Sectional Area of the Rockbolt
210 GPa	69.12 mm	380.13 mm^2^

## Data Availability

This article included all data.
